# Relative and Transport Efficiency-Independent Approach for the Determination of Nanoparticle Size Using Single-Particle ICP-MS

**DOI:** 10.1021/acs.analchem.3c01823

**Published:** 2023-06-27

**Authors:** Borja Moreira-Álvarez, Laura Cid-Barrio, Francisco Calderón-Celis, José M. Costa-Fernández, Jorge Ruiz Encinar

**Affiliations:** Department of Physical and Analytical Chemistry, University of Oviedo, Avenida Julian Claveria 8, 33006 Oviedo, Spain

## Abstract

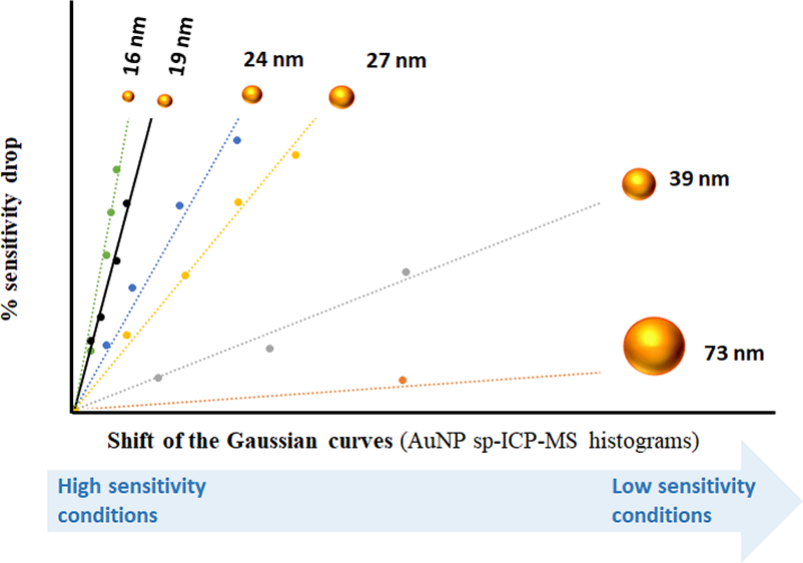

Herein, we introduce the first relative single-particle inductively coupled plasma mass spectrometry (spICP-MS) approach where size calibration is carried out using the target NP itself measured under different instrumental conditions without external dependence on the complex and prone-to-error determination of transport efficiency or mass flux calibrations, in contrast to most spICP-MS approaches. The simple approach proposed allows determining gold nanoparticle (AuNP) sizes, with errors ranging from 0.3 to 3.1% (corroborated by HR-TEM). It has been demonstrated that the changes observed in the single-particle histograms obtained for a suspension of AuNPs under different sensitivity conditions (*n* = 5) are directly and exclusively related to the mass (size) of the target AuNP itself. Interestingly, the relative nature of the approach shows that once the ICP-MS system has been calibrated with a generic NP standard, it is no longer necessary to repeat the calibration for the size determination of different unimetallic NPs carried out along time (at least 8 months), independently of their size (16–73 nm) and even nature (AuNP or AgNP). Additionally, neither the NP surface functionalization with biomolecules nor protein corona formation led to significant changes (relative errors slightly increased 1.3- to 1.5-fold, up to 7%) in the NP size determination, in contrast to conventional spICP-MS approaches where relative errors increased 2- to 8-fold, up to 32%. This feature could be especially valuable for the analysis of NPs in real samples without the need of matrix-matched calibration.

Applications of nanoparticles (NPs) have increased exponentially during the last decade in a wide range of different scientific and technological areas. While such nanomaterials are expected to provide many benefits, their routine use implies an accumulation of NPs in the environment, with a corresponding rise in the potential risk to environmental safety and human health. There is no clear understanding of the mechanisms, but the harmful effects of NPs may be closely related to a number of their properties including number concentration, size, surface chemistry, aggregation state, and how they interact with the environment.^1^

One of the most powerful analytical approaches for the detection and characterization of inorganic NPs dispersed in aqueous media is the use of inductively coupled plasma mass spectrometry,^2^ especially, when working in the single-particle mode (spICP-MS).^3,4^ This technique provides size, size distribution, and particle number concentration information from liquid samples containing inorganic NPs.^5^ A single dilution step such as sample pretreatment is required to guarantee that NPs reach the plasma individually, producing a single particle event (discrete pulse of ions) distinguishable from the continuous background.^6^ The frequency of the particle events can be then translated into particle number concentration. Moreover, the intensity of the signal obtained is proportional to the mass (particle size) of the target.^4−6^ Importantly, the incomplete transport of NPs from the nebulizer into the plasma is a key parameter that should be taken into consideration in order to guarantee accurate results.

The determination of nanoparticle size by spICP-MS has been attempted using a wide variety of methods. A straightforward approach is to generate a calibration curve of signal intensity versus particle diameter using well-characterized NP standards, containing the same element and with the same geometry and density of the target NP.^7^ However, such an approach is strongly limited by the availability of appropriate nanoparticle standards of different sizes.

This is why the more common methods used nowadays for NP size analysis by spICP-MS rely on the correlation between the signal intensity of dissolved inorganic standards and the mass of the NP. For this purpose, a mass flux curve obtained from the calibration of the corresponding dissolved inorganic standards is used to translate the measured intensity in the NP event into the mass of the metal present.^8^ One of the key terms that must be previously determined experimentally, unless it is known to be 100%, is the transport efficiency (TE), which has to be measured regularly (at least daily) to ensure the most accurate results. Measuring TE is far from being simple as it depends on a myriad of factors, including the components of the sample introduction system used (i.e., nebulizer type and status and the spray chamber), various operational parameters such as gas flow and uptake rates, and, of course, on the analyte type and matrix nature.^7,8^ Values reported in the literature typically range from 1 to 5% but can easily increase up to 50–90% for high-efficient nebulizers.^9^ Total consumption introduction systems allow to achieve almost quantitative TE values (>90%) but bring as inconvenience the use of delicate and expensive micronebulizers.^10^

Among the different strategies developed so far for TE determination, the waste collection of the waste stream exiting the spray chamber is an indirect way to determine the total analytes that enter the plasma by comparing the waste to the sample uptake volumes.^8^ However, such methods are not recommended as they typically suffer from strong biased results and require measuring the TE value regularly to ensure accurate results.

The most popular approaches for determining TE nowadays rely on the measurement of the signal pulse frequency of a standard NP suspension with a known particle number concentration.^11^ The ratio between the determined number of NPs reaching the plasma (number of signal events per second is equal to the number of NPs entering the plasma per second) and the number of NPs delivered to the nebulizer provides the TE. Here, the NP number concentration at the time of analysis should be accurately known. Additionally, this approach requires that eventual losses during transport to the plasma must be identical for both the NP standard and the inorganic dissolved standards that are later used for mass flux calibration.^8^

Alternatively, the ratio between the inorganic solution sensitivity (counts per nanogram of analyte delivered to the nebulizer, which is TE-dependent) and the NP sensitivity (counts per nanogram of analyte in an individual NP event, which is independent of the TE) can be used to determine the transport efficiency.^11^ This strategy requires the disposal of well-characterized NP standards. Additionally, the value of density of the NP is needed in this approach to compute the mass of the detectable element in a single NP.^12^ This density is often assumed to be the same as that of bulk materials, but very often the actual values in NPs are significantly lower, thus resulting in high errors in NP diameter calculations. Another assumption of this method is that the generation, extraction, and transport of ions from both a nanoparticle and dissolved inorganic ions are identical, which is not always the case.^13^ In that case, TE must include a correction factor that could vary among NPs, introducing an additional degree of uncertainty in the size analysis.

Regardless, the required NP standards must be measured every working day along with the target NPs which implies its constant availability in perfect conditions in the laboratory. Unfortunately, the availability of NP standards or RM is still very scarce and often is not certified for number concentration. Thus, the most typical AuNPs, AgNPs, or TiO_2_ NP standards are commonly used, assuming the same behavior as that of the target NP analyte, regardless of its size, composition, and chemical environment.^14^

A significant feature of spICP-MS is the capability to analyze NPs in complex samples with minimal sample preparation. However, interactions of NPs with the environment could affect the NP surface (e.g., protein corona formation when NPs are present in biological media), significantly compromising the accuracy of the measurement and requiring a matrix-matched calibration to minimize such effect.^15^ One of the most important handicaps is the impact of the sample matrix composition on the determination of the TE values.^4^ In fact, the composition of the sample matrix can influence differently the sensitivities from dissolved inorganic standards and the NPs,^11^ making it very difficult to decide whether to perform a matrix-matched calibration and how to do it.^16^

Therefore, despite the great potential and variety of approaches reported so far for NP size characterization using spICP-MS, all of them are critically dependent on the accurate determination of TE and/or the availability of adequate high-quality NP standards. Herein, we propose a conceptually new and simple approach that allows the reliable size determination of different-size AuNPs, with the average error of less than 3% difference from TEM determinations. Relative measurements carried out at different operational (sensitivity) conditions are enough to produce significant changes in the single-particle signals that are directly related with the mass (size) of the target NP and completely independent both of the TE value of the nebulization system used and, above all, of the biomolecules that are intentionally (conjugated) or accidentally (matrix) attached to their surface. Interestingly, the relative nature of the measurements shows that the calibration curve built once with a generic NP standard/RM can be used over time for any other unimetal target NP, independently of their size, nature, surface functionalization, and matrix.

## Experimental Section

### Reagents, Solutions, and Materials

Dissolved inorganic Au standards were prepared from a 1000 mg L^–1^ Au standard (Merck, Germany). Hydrogen tetrachloroaurate trihydrate, sodium citrate tribasic dihydrate, bovine serum albumin (BSA), and Tween-20 were purchased from Sigma-Aldrich (St. Louis, USA). Deionized ultrapure water (18.2 MΩ·cm) was obtained with a PURE LAB flex3 apparatus (ELGA Labwater, UK). AuNPs with diameters of 16.7 and 27.4 nm were synthesized in our laboratory following a well-established procedure described elsewhere.^17^

Commercial AuNPs (39.6 nm) and AgNPs (40.1 and 51.3 nm) were purchased from Nanocomposix (San Diego, USA), while Au NPs of 18.7 nm (free and conjugated to avidin), 24.4 nm (free and conjugated to PEG), and 73.3 nm diameter were purchased from Nanovex (Oviedo, Spain). Detailed information of the surface-functionalized AuNPs is given in the Supporting Information.

### Instrumentation

All work was performed with an ICP-MS/MS system equipped with a Micromist nebulizer and a 1.5 mm ID quartz torch injector (Agilent Technologies, CA, USA). The ICP-MS/MS settings and operating conditions are listed in Table S1. The spICP-MS NP analysis was carried out using 5 ms of dwell time. Method validation was carried out by the comparison of the here-developed approach with the results of the NP analysis obtained using the accessible spreadsheet developed by Wageningen Food Safety Research, commonly known as RIKILT.^18^

## Results and Discussion

**Figure 1:**
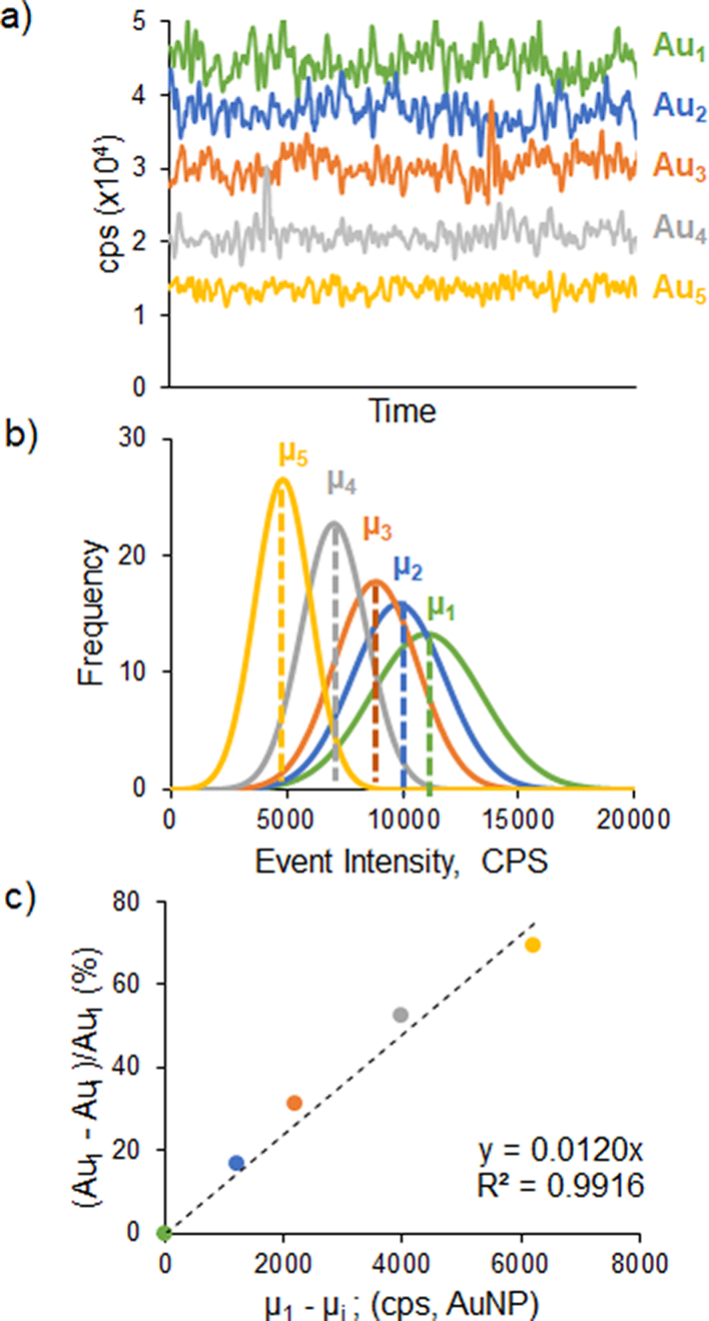
Workflow employed to correlate the (c) relative drop in signal intensity observed for the 1 ppb Au ionic standard (a) when operating the ICP-MS instrument under conditions of decreasing order of sensitivity (from 1, the highest, to 5, the lowest) with the left shift of the median values (b) of the corresponding histograms of the sp events obtained for a AuNP of 24.4 nm.

The transport efficiency-independent spICP-MS strategy developed, schematically illustrated in Figure 1, allows to correlate the AuNP size (mass) with the variation of the intensity and frequency of the particle events registered for the same NP solution analyzed under different (*n* = 5) sensitivity conditions. The step-by-step variation of the lens voltages of ICP-MS led to a global sensitivity drop of around 50–60%, as can be clearly seen in Figure 1a for the continuous measurement of a Au inorganic standard under each instrumental condition. Such sensitivity variations resulted in significant changes in the typical Gaussian fit of the corresponding histograms (frequency vs cps/event) obtained from the single-particle events of the NP sample measured (Figure 1b). In fact, as can be seen in Figure 1c, the drop in sensitivity (%) of the Au inorganic standard signal observed when changing the instrumental conditions correlated very well (*R*^2^ > 0.99) with the shifts to lower values of the median of the different Gaussian fits (sp events) obtained for the target NP solution measured under the same conditions. The relative nature of the measurement makes this approach robust and generic, independent of the NP size, nature (e.g., Au and Ag), NP surface functionalization, and matrix composition. It is clear that an adequate data treatment of the sp measurements carried out under the different instrumental conditions is required in order to obtain an accurate determination of the slope of the linear correlation curve mentioned above and thus will be explained below.

### Data Treatment

**Figure 2:**
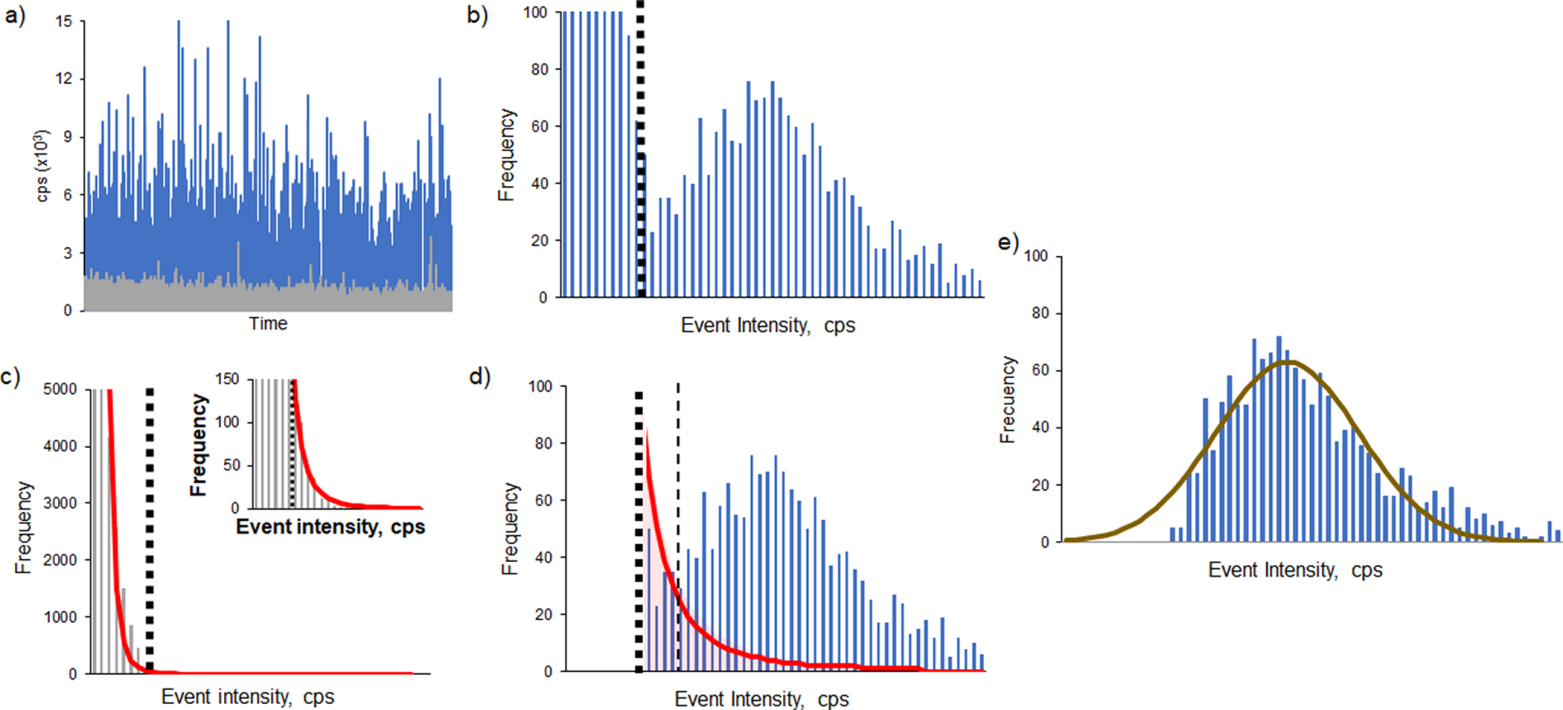
Data treatment of the sp-ICP-MS signals obtained for the AuNPs of 18.7 nm. The events obtained for the AuNP and the corresponding blank (a, blue and gray, respectively) are sorted to obtain histograms according to their frequency (b and c, respectively). The histogram of the blank was fitted to an exponential curve (red trace, c and d) that was then applied to the NP sample histogram to subtract the blank contribution and to obtain the definitive Gaussian curve (e). Dotted and dashed lines (b–d) correspond to the threshold values of 3σ and 5σ of the blank.

spICP-MS signals obtained were treated using the procedure shown in Figure 2. The data collected for sample analysis (AuNPs, 18.7 nm diameter) and the corresponding blank signals (blue and gray events in Figure 2a, respectively) were sorted to achieve a histogram of frequency distribution of each event for the blank (gray histogram, Figure 2c) and AuNPs (blue histogram, Figure 2b). Removal of the ionic background contribution from the sample, so that only nanoparticle-related events remain in the NP histogram, is critical in the here-developed strategy since the sample NP histograms obtained at low-sensitivity conditions (yellow trace in Figure 1) approach the blank histogram. The procedure developed for this purpose consists of two steps. The values of the event intensities from the measurement of the blank were sorted to determine the median (*y*_m_) and the standard deviation (σ) at first. The median was chosen instead of the mean because it is a better midpoint metric less affected by the presence of outliers or spikes. According to the criteria of three times the standard deviation (3σ),^19,20^ any signal below *y*_m_ + 3σ is considered as an effect of instrumental background and therefore neglected (black dotted line in Figure 2b–d). However, as shown in the inset to Figure 2c, there are still blank events outside this range that could interfere with the final histogram of the sample NPs. In fact, the histogram of the blank clearly shows an exponential decay (red curve in Figure 2c,d). This trend was observed for every blank measured, including different solutions (ultrapure water, 2% HNO_3_, and 2% HCl). The exponential curve obtained fitted very well to the blank experimental data (>96%) and could be then applied to subtract the ionic contribution (red shadow below the red curve in Figure 2d)^4,21^ beyond the *y*_m_ + 3σ threshold to the NP sample histogram and this way correct better the overestimation of the corresponding NP frequencies due to the blank contribution. The definitive NP sample histograms obtained (Figure 2e) at the different sensitivity conditions could be then fitted to Gaussian curves in order to get their corresponding median values and standard deviations (Figure 1b).

There is no universally accepted methodology that would allow an objective distinction of the contribution of the events resulting from the free ions present in the blank and the NP sample. One of the most widespread strategies makes use of a minimum threshold value of the event intensity distribution that can be computed as a multiple (from 3 to the most stringent 5 or even 7)^20^ of the standard deviation of the continuous (blank) signal. This is a very simple and fast strategy but could lead to under- or overestimations of the NP signals, especially when target NPs are close to the detection limit of the technique.^22^ In our case, we have observed still a blank contribution after the application of the stringent 5σ criterion (red-shadowed area after the thin dashed line in Figure 2d) in those curves obtained for the small NPs (<25 nm) under the low-sensitivity conditions. Other authors have resorted to algorithms to classify events as a function of their origin or to signal deconvolution procedures to better discriminate the blank contribution.^21,23^ These approaches lead to good results but are sample-specific and complex. Their application in our case would be very complex as our concept is based on the measurement of the NP sample under different sensitivity conditions. The data treatment proposed here is a compromise procedure that complements the simplicity of the 3σ threshold with the finer exponential curve that fitted very well to the remaining blank events, thereby allowing to compensate its contribution to the event distribution in the NP sample. Notably, this procedure is applied automatically without any customization to the individual measurements of the same NP sample.

### Method Development

**Figure 3:**
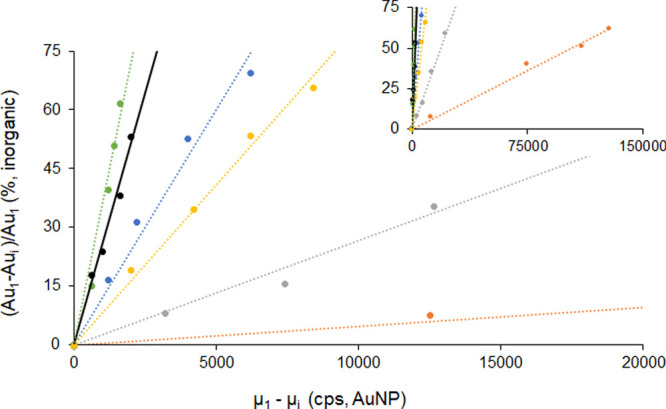
Linear correlation between the signal drop for Au ionic standard and the shift of the median values of the Gaussian curves fitted to the corresponding AuNP histograms obtained under the same conditions of declining sensitivity. Six curves are plotted corresponding to different AuNPs: 16.4 nm (green, *R*^2^ = 0.991), 18.7 nm (black, *R*^2^ = 0.996), 24.4 nm (blue, *R*^2^ = 0.991), 27.4 nm (yellow, *R*^2^ = 0.997), 39.6 nm (gray, *R*^2^ = 0.993), and 73.3 nm (red, *R*^2^ = 0.997).

We first noted that the intensity and frequency of the events obtained for the spICP-MS analysis of a given AuNP sample under different sensitivity conditions were significantly different. The typical Gaussian curves of the corresponding sp event histograms in particular became narrower, and their median value (μ_*i*_) shifted to lower sensitivity (cps/event) values (Figure 1b) when instrumental parameters were modified to low-sensitivity conditions. This behavior was somehow expected since the ability to discriminate between NP populations with a few atoms of difference declines with the sensitivity of the detection system used, which leads to detect with the same and lower intensity (cps/event), and thus higher frequency, NP populations that could be distinguished with higher sensitivity. Our hypothesis was that the shift of the median values obtained at lower sensitivities with regard to the median value obtained under the highest sensitivity conditions could be closely related to the number of atoms (mass) present in the NP under study and, therefore, specific to each NP mass (size). In order to make this feature of each NP size independent of the starting instrumental sensitivity observed each day, we normalized the sensitivity drop by measuring a 1 ppb solution of an ICP-MS Au standard under the same instrumental conditions (Figure 1a). The decrease in the sensitivity (%) for the Au inorganic standard caused by the instrumental changes correlated very well (*R*^2^ ≥ 0.99) with the shifts to lower values of the median of the different Gaussian signal events obtained for the target NP sample measured under the same instrumental conditions (Figure 1c), the slope (*m*) of such linear correlation observed being highly specific for each NP size/mass assayed. As an example, Figure 1 shows the scheme of the workflow obtained for the determination of the specific slope corresponding to a sample containing AuNPs of 24.4 nm diameter. The final demonstration of the unequivocal relationship between the slope of the linear correlation and the size/mass of the corresponding AuNP sample studied is given in Figure 3. Up to six different AuNP samples with sizes ranging from 16.4 to 73.3 nm provided significantly different linear correlation curves with statistically different slopes (see Table 1) and excellent correlation coefficients (*R*^2^ ranging from 0.991 to 0.997). In fact, two AuNPs of very similar size, 16.4 and 18.7 nm, could be clearly discriminated at the 95% confidence interval by their different slopes (0.0360 ± 0.0017 and 0.0256 ± 0.0008, respectively).

**Table 1:** NP Diameters (nm) and Slopes of the Corresponding Correlation Curves Experimentally Obtained for the Five Different AuNPs Shown in Figure 3^a^

TEM^b^	slope ± 2 SD	this approach^c^	error (%)
18.7 ± 0.3	0.0256 ± 0.0008		
16.4 ± 0.3	0.0360 ± 0.0017	16.7 ± 0.7	1.8
24.4 ± 0.3	0.0120 ± 0.0006	24.1 ± 1.0	1.2
27.4 ± 0.5	0.00817 ± 0.00022	27.3 ± 0.9	0.4
39.6 ± 0.7	0.00267 ± 0.00009	39.7 ± 1.3	0.3
73.3 ± 1.3	0.000469 ± 0.000013	71.0 ± 2.2	3.1

a The AuNP of 18.7 nm was measured the same working day and used as the reference standard. Relative errors (%) are computed with respect to TEM values.

b Uncertainty corresponds to the standard error of the mean (*k* = 2) of the TEM images.

c Combined standard uncertainty (95% confidence interval).

Once it is verified that the slopes of the curves plotted in Figure 3 are highly specific of the mass/volume of the different AuNPs analyzed, we could assume that the ratio between their slopes (*m_i_*) and their corresponding NP volumes (*V_i_*) would be constant (α). Therefore, as shown in eq 1, we could use the slopes (and their uncertainty) experimentally obtained using the strategy developed for one AuNP standard (*m*_std_) and one unknown AuNP (*m_x_*) to compute α, which could be later used to determine accurately the volume of the unknown AuNP (*V_x_*) as the volume of the AuNP standard (*V*_std_) and its associated uncertainty are precisely known.
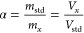
1Assuming a spherical shape, the volume and associated uncertainty of the unknown and standard AuNPs can be easily translated into the diameter and its associated uncertainty. Table 1 shows the values of slopes and diameters experimentally obtained for the five different AuNPs shown in Figure 3, using the AuNP of 18.7 nm as the reference standard. As can be seen, every NP diameter obtained agrees very well with the nominal values obtained by the TEM analysis (Figures S1–S6) with low relative errors ranging from 0.4 to 3.1%. Results for the bigger NPs were confirmed as well using the nanoparticle tracking analysis (NTA). The combined uncertainty ranged from 3 to 4% (95% confidence, *k* = 2), with similar contributions from the uncertainties associated to the variations in the volume of the AuNP standard (2.1%, calculated from its diameter) and the slopes experimentally obtained. It should be noted that the precise results given in Table 1 correspond to the nominal mean diameters of the unknown AuNPs and the uncertainty associated to their determination using our approach. In order to know the dispersion of the population of the different AuNPs present in each solution, we used both the nominal value (Table 1) and the fitted Gaussian curve (described in the Data Treatment section and shown in Figure 2e) obtained under maximum sensitivity conditions. Dispersion intervals (1 SD) obtained are given in Table S2 which ranged from 8.4 to 17% RSD, similar to those observed by TEM (11–14% RSD).

It is worth stressing that cumbersome and prone-to-error determination of TE is not necessary anymore. In order to support this claim, we compared the results obtained using the regular concentric nebulizer with low TE (2.5%) used throughout this work with those obtained using a total consumption nebulizer with high TE (100%) for three AuNPs (16.4, 27.4, and 39.6 nm). As can be seen in Table S3, the relative errors obtained ranged between 0.7 and 1.5%, which is very similar to the error range obtained using the regular nebulizer, 0.3–1.8% (see Table 1). This is an important advantage of the here-proposed approach, as TE is a specific and extremely variable factor that needs to be measured daily in most conventional sp approaches to ensure the most accurate results. Furthermore, the relative nature of the method developed make it reproducible and independent of the working day when the slopes of the standard and unknown AuNPs (*m*_std_ and *m_x_*, respectively) are measured. In fact, *m*_std_ could be determined once and used for the quantification of unknown AuNPs along time without the need to measure the AuNP standard within the same working day, as it is the case for standard sp approaches. Aiming at proving such a hypothesis, *m*_std_ obtained on a certain day for the 18.7 nm AuNP standard (see Table 1) was later applied to determine the diameter of the five different AuNPs measured on seven different days over a period of 8 months. As can be seen in Table 2, the results obtained agreed again very well with the corresponding TEM values (relative errors ranging from 0.24 to 4.5%) and those obtained when the NP standard was measured within the same working day (Table 1). In fact, due to the high consistency of the data obtained over time, the combined uncertainty shown in Table 2, which takes into account not only the individual uncertainties but also the reproducibility of the seven replicates performed in different working days (*n* = 7), is still low (ranging from 6 to 10%, *k* = 2) and only double the combined uncertainty given in Table 1 for a set of individual experiments. Such results evidence that this strategy does not require for measuring a similar AuNP standard every working day along with the target NPs, which would imply its constant availability in perfect conditions in the laboratory.

**Table 2:** Relative Error and Mean of the Replicates Experimentally Obtained Using Both the Approach Developed and the Regular sp Approach over 8 Months for Five Different AuNPs (*n* = 7) and One AgNP (*n* = 5, Last Row)^a^

diameter TEM^b^	this approach		regular sp	
	diameter^c^	error (%)	diameter^c^	error (%)
16.4 ± 0.3	16.6 ± 1.2	1.2	16.3 ± 1.2	0.61
24.4 ± 0.3	24.7 ± 1.6	1.1	23.2 ± 1.5	4.9
27.4 ± 0.5	27.1 ± 1.7	1.0	25.5 ± 1.7	6.9
39.6 ± 0.7	39.5 ± 2.8	0.24	41.0 ± 3.1	3.5
73.3 ± 1.3	70.0 ± 7.4	4.5	61.9 ± 7.6	16
			(68.1 ± 8.0)^d^	(7.1)^d^
40.1 ± 0.6 (AgNP)	39.5 ± 3.1	1.5	51.7 ± 3.2	29
			(39.3 ± 5.1)^d^	(2.0)^d^

a The reference standard (AuNP, 18.7 nm) was measured once at the beginning of the time period and every working day for the developed and the regular sp approach, respectively.

b Uncertainty corresponds to the standard error of the mean (*k* = 2) of the TEM images.

c Combined standard uncertainty comprising individual uncertainty and reproducibility of the *n* replicates (95% confidence interval).

d Results obtained when using AuNPs of 39.6 nm for the AuNPs of 73.3 nm and AgNPs of 51.3 nm for the AgNPs of 40.1 nm as reference standards, respectively.

Although the integration time used in this work is quite high (5 ms) in order to make the approach accessible to any ICP-MS instrument, the advantages of the use of lower dwell times in sp analysis have been widely reported, especially for smaller NPs.^11,12,20^ Thus, three AuNPs (16.4, 24.4, and 39.6 nm) were measured at five different dwell times (5 ms, 3 ms, 1 ms, 500 μs, and 100 μs). As can be clearly seen in Tables S4–S6, the results obtained were statistically indistinguishable with those originally obtained using 5 ms in terms of accuracy and precision and demonstrate that our approach is fully compatible with high and low dwell times. We wanted to assess as well the influence of the particle concentration. For that purpose, two AuNPs (27.4 and 39.6 nm) were measured at higher concentration levels (2× and 3×). The results shown in Table S7 demonstrate that nanoparticle concentration seems not to play a critical role in terms of accuracy; as expected, however, it seems that precision deteriorates slightly as the concentration increases. Therefore, as it is the case in most of the sp-ICPMS-based approaches,^4,11^ we recommend to work at low concentration levels.

We then assessed whether *m*_std_ obtained for the 18.7 nm AuNP standard could be further applied to determine the diameter of NPs of a different nature and size (e.g., AgNP of 40.1 nm diameter). As opposed to Au that is monoisotopic (197), Ag presents two isotopes, 107 and 109. We selected 107 because of its slightly higher isotopic abundance (52%). The same modification on the sensitivity conditions was performed, but, in this case, we resorted to an inorganic Ag standard to normalize the sensitivity changes (Figure 1a). Then, we performed the correlation of the sensitivity drops (%) observed for the Ag inorganic standard with the shifts to lower values of the median of the different Gaussian curves obtained for the target AgNP solution measured under the same instrumental conditions (Figure 1c) to compute the corresponding (*m_x_*). As the slope of the AuNP standard (*m*_std_) was obtained from a different element, it was necessary to take into account the isotopic abundance of the measured Ag isotope in order to compute α (eq 1) and then the volume of the unknown AgNP (*V_x_*). The final diameter obtained as the average of five replicates carried out along time (5 months) was 39.5 ± 3.1 nm (*k* = 2), again in excellent agreement with the nominal TEM value (1.5% error). The combined uncertainty (8% RSD, *n* = 5) was also similar to those obtained for the NP of the same element (Au) shown in Table 2.

### Critical Comparison to the Standard sp Approach

The same set of sp data (*n* = 7) shown in Table 2 used to obtain the AuNP diameters throughout the assessment period (8 months) was also treated using the regular sp approach (RIKILT spreadsheet) for comparison purposes.^18^ In this case, as required for the regular sp approach, the AuNP of 18.7 nm was measured on each of the 7 days of analysis for TE computation. The relative errors obtained, with regard to the TEM reference values, for the small- and medium-sized AuNPs (up to 40 nm) were significantly higher using the regular sp approach (mean error, 4%) than the proposed approach (mean error, 0.9%). The relative error skyrocketed to 16% when the size of the target AuNP (73 nm) was much higher than that of the NP standard. This error is even higher (29%) when the element that forms the NP is different in the target (Ag) than in the standard (Au). Notably, in these two last cases, the error obtained using the approach proposed herein is much lower (4.5 and 1.5%, respectively) and similar to those obtained for the small- and medium-sized AuNPs. The accuracy of regular sp approaches is critically dependent on the assumption that TE computed for the NP standard is the same for the target NP, which is not the case when they differ greatly in size or nature. This is why the use of NP standards of the same composition and similar size in regular sp approaches has been recently recommended.^14^ In fact, Table 2 shows that when using AuNPs of 39.6 nm and AgNPs of 51.3 nm as reference standards for the size determination of bigger AuNPs and AgNPs, the regular sp results obtained (68.1 ± 8.0, error 7.1% and 39.3 ± 5.1 nm, error 2.0%, respectively) were much closer to the TEM values and matched pretty well with our data. Therefore, the independency of the approach proposed upon the TE makes it much more reliable and accurate, particularly as the differences between the target and standard NPs are more significant.

### Application to Surface-Functionalized AuNPs

We finally wanted to assess if the relative nature of the approach developed make it suitable for the analysis of surface-modified NPs. We first selected a small (3 kDa) thiolated PEG carboxylated molecule and the AuNP of 24.4 nm. Therefore, only the size of the functionalized AuNP was slightly increased in comparison to the original (“free”) AuNP. Table 3 shows that the impact of such small increase was negligible on the accuracy of the NP sizes obtained using both the proposed and regular sp approaches, in line with the results recently published by Montoro Bustos et al.^24^ We later selected avidin, a tetrameric binding protein (68 kDa), to functionalize the AuNP of 18.7 nm. Again, the impact of the chemical functionalization was negligible on the AuNP diameter obtained using the proposed approach but already noticeable (relative error increased from 3 to 9%) when using the regular sp approach.

**Table 3:** Effect of Surface Functionalization and Protein Corona Formation on the AuNP Diameter (nm) Experimentally Obtained Using Both the Approach Developed and the Regular sp Approach^a^

TEM	surface modification	this approach	regular sp
18.7	free	19.0 ± 0.8 (1.6%)	19.3 ± 0.8 (3.2%)
	conjugation (avidin)	18.8 ± 1.1 (0.5%)	20.3 ± 1.1 (8.6%)
	protein corona	19.1 ± 1.6 (2.1%)	23.1 ± 1.6 (24%)
24.4	free	24.8 ± 1.7 (1.6%)	24.1 ± 1.7 (1.2%)
	conjugation (PEG)	24.9 ± 2.2 (2.1%)	25.8 ± 2.7 (5.7%)
39.6	free	40.1 ± 2.7 (1.3%)	42.2 ± 2.8 (6.6%)
	protein corona	40.4 ± 3.2 (2.0%)	35.3 ± 4.6 (11%)
73.3	free	70.1 ± 7.3 (4.4%)	63.5 ± 7.0 (13%)
	protein corona	68.3 ± 8.0 (6.8%)	50.1 ± 12.5 (32%)

a Relative error (%) compared to the TEM values are given in brackets. Uncertainty corresponds to 95% confidence interval (*k* = 2).

NPs present in biological samples can be rapidly covered by a selected group of biomolecules (mostly proteins) to form the so-called “protein corona”, which determines the way they interact with biological systems and their final metabolic fate. It is well known that the number of proteins that could attach to NPs in such biological media can be high and well over 100.^25^ This modification greatly changes the NP size and nature of its surface, and therefore its TE, leading to significant errors when assessing the size of unknown NPs in biological samples by regular sp approaches. In order to evaluate the potential of our approach to overcome this limitation, thanks to its independency of the TE, we incubated AuNPs of different sizes (18.7, 39.6, and 73.3 nm) with a high excess of protein (fixed BSA:Au mass ratio of 9500). Results are also collected in Table 3. As expected, the protein corona affected severely the size determination obtained by the regular sp approach. Ranges of relative error increased from 3–13% when “free” to 11–32% when surrounded by proteins. Precision also worsens substantially, especially for the bigger AuNPs (almost a factor of 2). In contrast, we did not find significant changes when measuring free and protein-covered AuNPs using our approach. In spite of the protein shell, ranges of relative error remained constant, from 1.6–4.4% without protein corona to 2.0–6.8% with protein corona. In addition, the combined uncertainty remained rather steady and similar to that observed for the corresponding free AuNPs.

## Conclusions

An innovative strategy for the size determination of metallic NPs has been successfully developed that relies exclusively on the shifts to lower values of the Gaussian curves fitting the spICP-MS histograms observed for the same NP when lowering the sensitivity. After normalization by the relative sensitivity drop observed for the ionic standard under the same instrumental conditions, highly linear relationships (*R*^2^ > 0.99) are observed, whose slopes are highly specific to the mass (size) of the NP analyzed. The slope ratios of two NP solutions are found to be equivalent to their volume ratio, so that if one well-characterized NP standard is taken as the reference, the size of the other NP can be directly and precisely determined. Such standard NP can be measured once and used as reference over time without compromising the accuracy and precision. This approach shows some advantages over the established spICP-MS methods. With regard to the size calibration using NP standards, the relative nature of our approach shows that we just need to use a single NP standard, once in time and not necessarily very similar to the target NP without the need to compute TE. This is a major advantage, especially due to the shortage of well-characterized and stable NP standards of different types. Concerning the size calibration methods using ionic standard solutions and TE determination, on the one hand, we do not need an NP standard certified in particle number concentration. On the other hand, we do not need to know the NP density as we do not translate signal intensity into NP mass using a mass flow calibration built upon ionic standards. Changes in ionization and ion extraction and transmission neither affect us. Last but not least, the approach proposed is not impacted by differential matrix effects on ionic and NP sensitivities.

Although this approach has been developed for AuNPs, it is still general, with great potential to be applied to other unimetallic NPs whose metallic element is different from that of the NP standard. It has been already proved for AgNPs, but eventually, it could also be valid for Pt, Pd, or Cu NPs. Notably, the relative nature of the approach makes it independent from the molecules intentionally (conjugated) or accidentally (matrix) attached to the NP surface as well. In this sense, we can clearly envisage its application as a diagnostic tool for the assessment of unknown NPs in real (biological, food, and environmental) samples, where there is no control or knowledge about the NP surface interactions, without the need for measuring a matrix-matched NP standard within the same working day.

## Supporting Information Available

TEM images of the different nanoparticles employed, description of the methods employed for surface modifications of AuNPs, and operating conditions and additional experimental results (PDF)
