# Supplementary material for: Relative and Transport Efficiency-Independent Approach for the Determination of Nanoparticle Size Using Single-Particle ICP-MS
Source: Anal Chem. 2023 Jun 27;95(27):10430–7. doi: 10.1021/acs.analchem.3c01823 (PMC10339277; doi:10.1021/acs.analchem.3c01823)
Supplement: Supplementary file 1 — ac3c01823_si_001.pdf [file ac3c01823_si_001.pdf]

Supporting Information

Relative and transport efficiency-independent approach for the determination of nanoparticle size using single particle ICP-MS

Borja Moreira-Álvarez, Laura Cid-Barrio, Francisco Calderon-Celis, José M. Costa-Fernández and Jorge Ruiz Encinar**

Department of Physical and Analytical Chemistry, University of Oviedo, Avenida Julian Claveria 8, 33006, Oviedo, Spain

* *Jose M Costa-Fernandez: jcostafe@uniovi.es*

* *Jorge Ruiz Encinar: ruizjorge@uniovi.es*

Table of Contents

Figure S1. TEM image and size distribution histogram (n=192) of 16 nm synthesized AuNPs.............................S3

Figure S2. TEM image and size distribution histogram (n=205) of 18nm commercial AuNPs. S3

Figure S3. TEM image and size distribution histogram (n=197) of 25nm commercial AuNPs. S3

Figure S4. TEM image and size distribution histogram (n=209) of 27nm synthesized AuNPs..............................S4

Figure S5. Size characterization of 40 nm commercial AuNPs by (a) TEM image with the size distribution histogram (n=208) obtained from TEM measurements and (b) NTA size distribution histogram (n=1069). S4

Figure S6. Size characterization of 70 nm commercial AuNPs by (a) TEM image with the size distribution histogram (n=216) obtained from TEM measurements and (b) NTA size distribution histogram (n=2300). S5

Figure S7. Size characterization of 40 nm commercial AgNPs by (a) TEM image with the size distribution histogram (n=200) obtained from TEM measurements and (b) NTA size distribution histogram (n=1746). S6

Table S1. ICP-MS/MS settings and operating conditions S6

Table S2. Size distributions (diameters, nm) obtained for different AuNPs using the nominal values (Table 1) and the fitted Gaussian curves (sp-ICP-MS histograms) obtained under maximum sensitivity conditions. S7

Table S3. Comparison of the relative errors and mean of the replicates experimentally obtained with the concentric nebulizer with low TE (2.5%) used throughout the work and a Total Consumption Nebulizer (TE 100%). S7

Table S4. Relative error and mean of the replicates experimentally obtained using the approach developed for the 39.6 nm AuNPs using different dwell times...S7

Table S5. Relative error and mean of the replicates experimentally obtained using the approach developed for the 24.4 nm AuNPs using different dwell times...S8

Table S6. Relative error and mean of the replicates experimentally obtained using the approach developed for the 16.7 nm AuNPs using different dwell times...S8

Table S7. Mean of the three replicates experimentally obtained using the approach developed at different concentration levels. S8

Description of Surface functionalization of AuNPs...S9

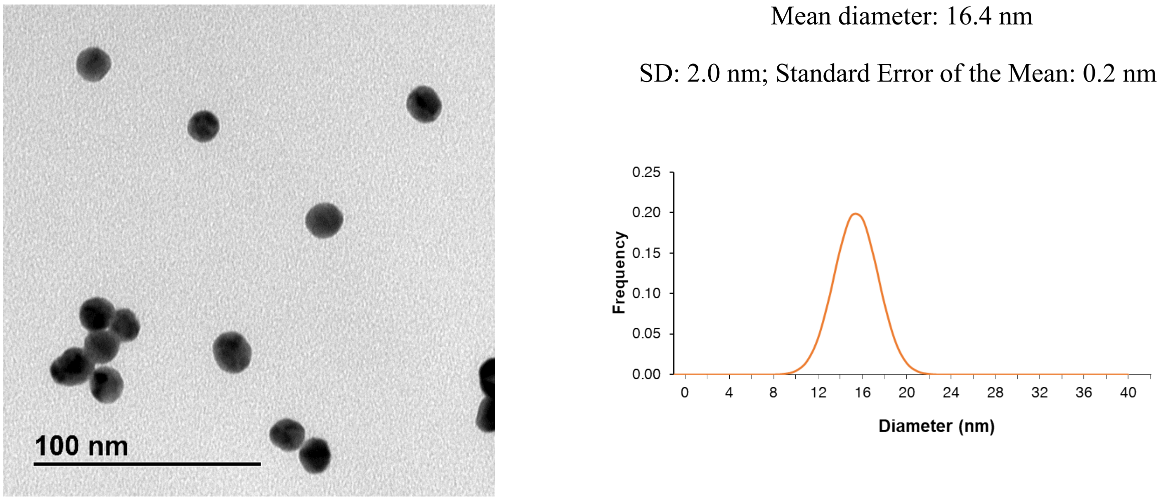

Figure S1. TEM image and size distribution histogram (n=192) of 16 nm synthesized AuNPs.

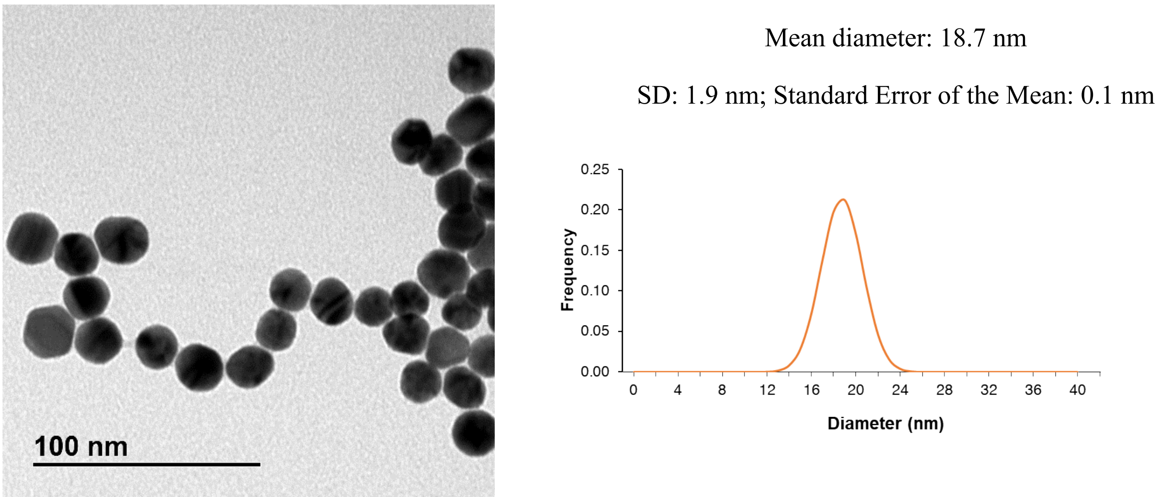

Figure S2. TEM image and size distribution histogram (n=205) of 18nm commercial AuNPs.

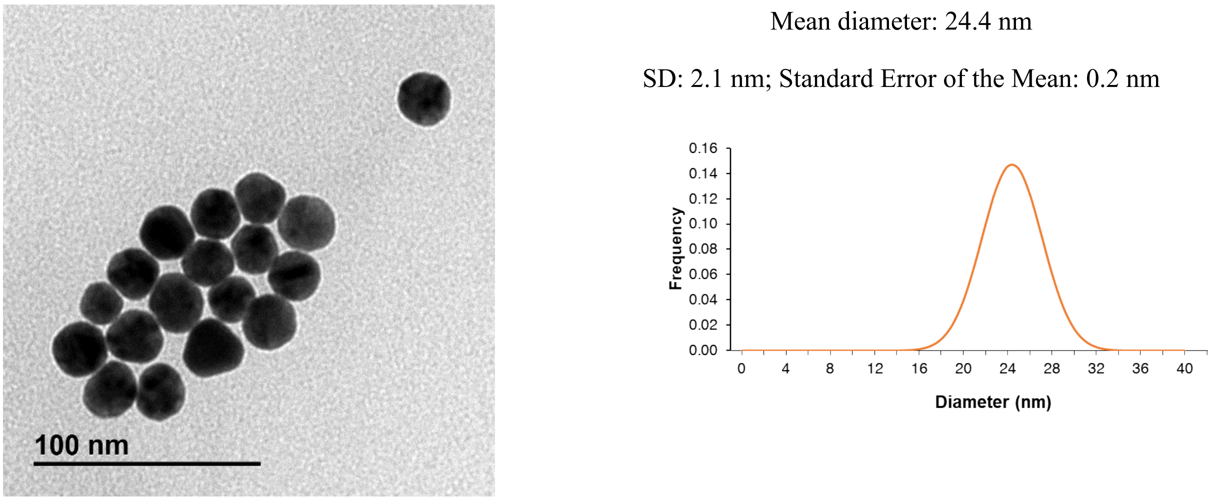

Figure S3. TEM image and size distribution histogram (n=197) of 25nm commercial AuNPs.

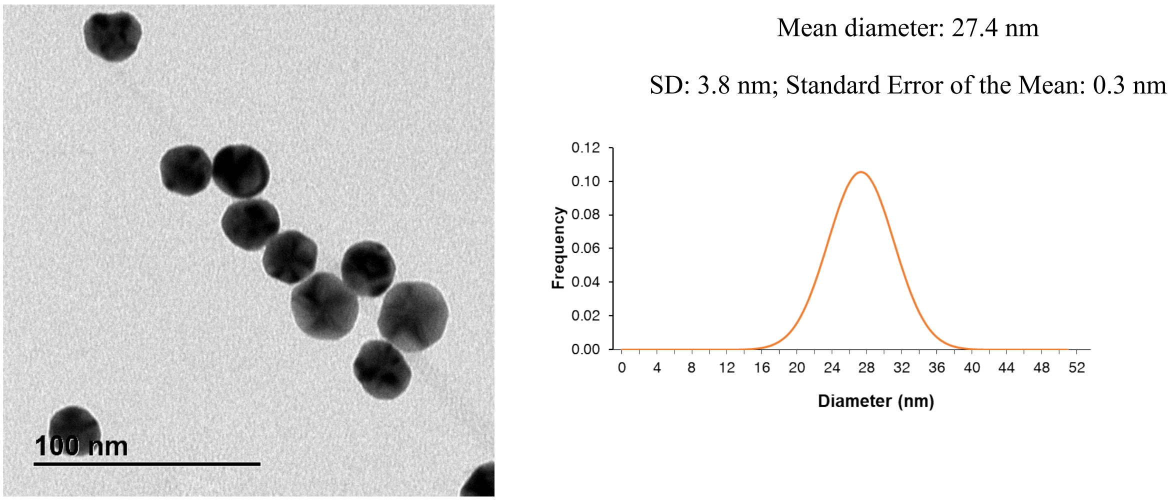

Figure S4. TEM image and size distribution histogram (n=209) of 27nm synthesized AuNPs.

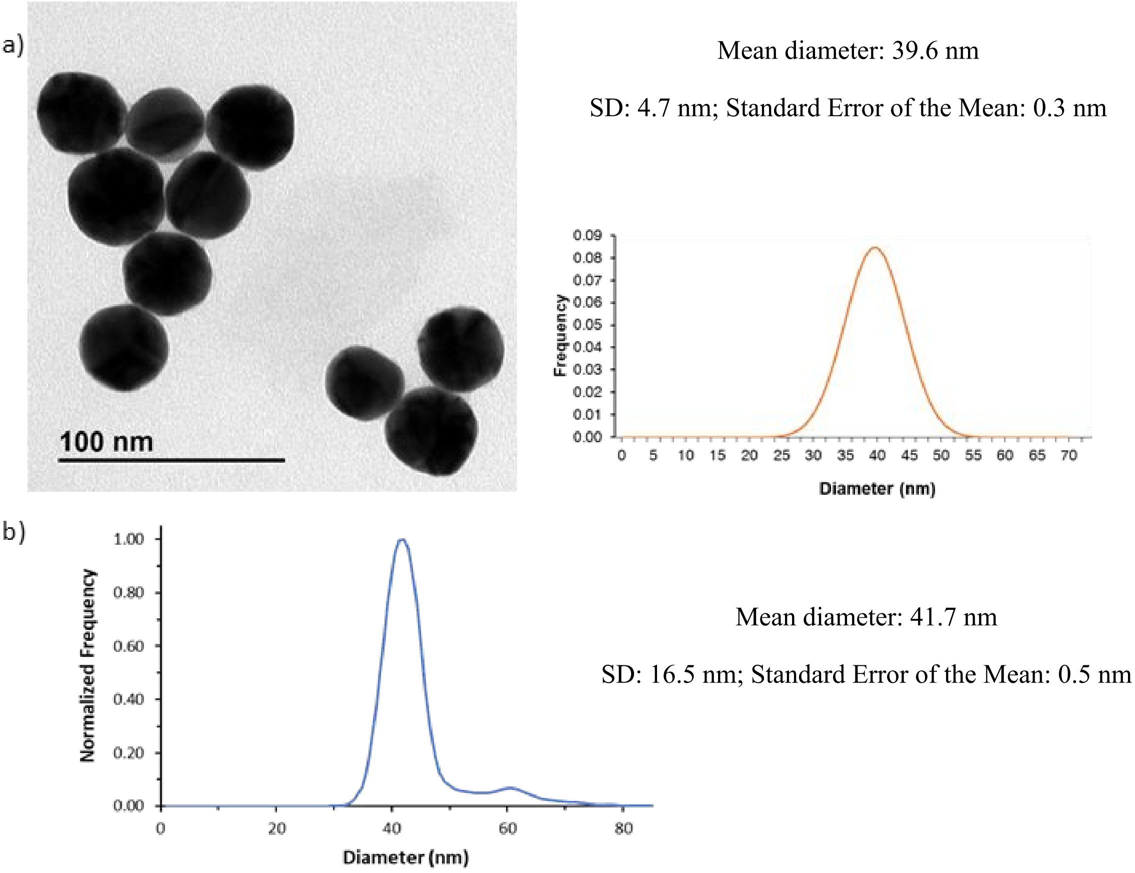

Figure S5. Size characterization of 40 nm commercial AuNPs by (a) TEM image with the size distribution histogram (n=208) obtained from TEM measurements and (b) NTA size distribution histogram (n=1069).

a)

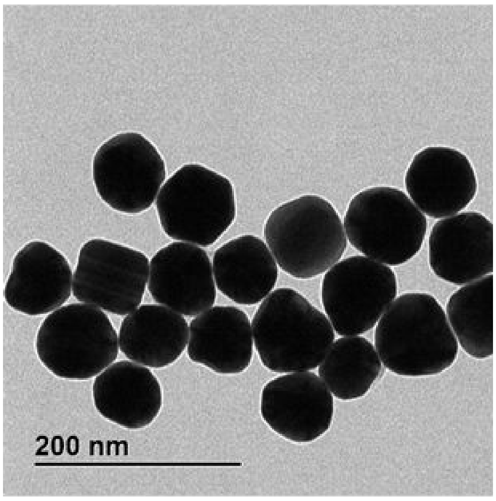

Mean diameter: 73.3 nm

SD: 9.6 nm; Standard Error of the Mean: 0.7 nm

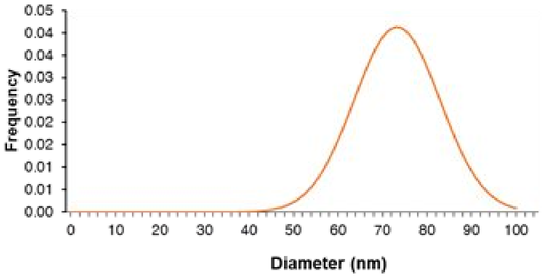

b)

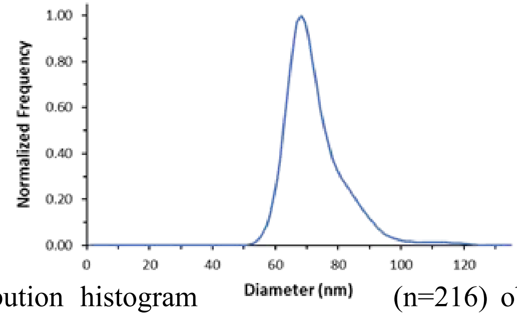

Mean diameter: 73.3 nm

SD: 17.2 nm; Standard Error of the Mean: 0.4 nm

Figure S6. Size characterization of 70 nm commercial AuNPs by (a) TEM image with the size distribution histogram (n=216) obtained from TEM measurements and (b) NTA size distribution histogram (n=2300).

a)

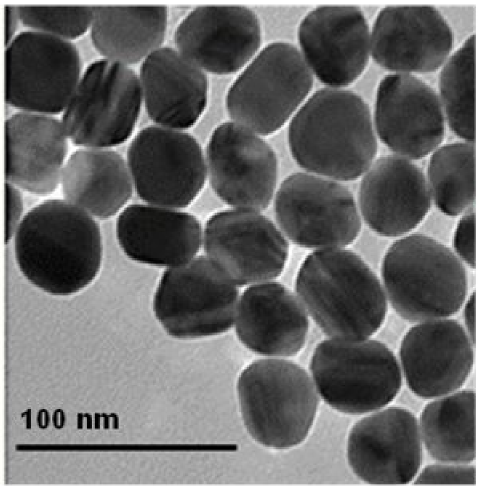

Mean diameter: 40.1 nm

SD: 4.1 nm; Standard Error of the Mean: 0.3 nm

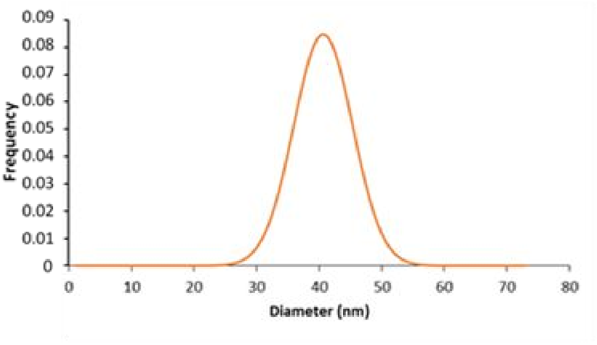

b)

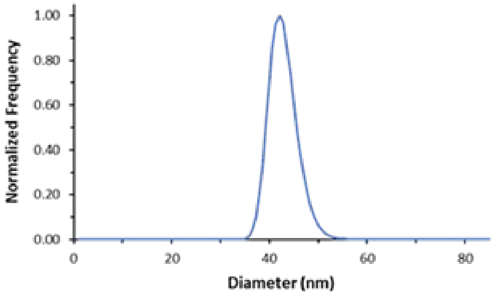

Mean diameter: 43.3 nm

SD: 8.1 nm; Standard Error of the Mean: 0.2 nm

Figure S7. Size characterization of 40 nm commercial AgNPs by (a) TEM image with the size distribution histogram (n=200) obtained from TEM measurements and (b) NTA size distribution histogram (n=1746).

Table S1. ICP-MS/MS settings and operating conditions

Plasma parameters	RF Power	1550 W
	RF Matching	1.25 V
	Sample Depth	7.0 mm
	Nebulizer Gas (Ar)	0.75 L/min
	Makeup Gas (Ar)	0.1 L/min
Lens	Extract 1	-12.0 V
	Extract 2	-165.0 V
	Omega Bias	-80 V
	Omega Lens	Variable
Detector	^{197}Au -> ^{197}Au	“on mass”
	Dwell Time	5, 3, 1, 0.5, 0.1 ms

Table S2. Size distributions (diameters, nm) obtained for different AuNPs using the nominal values (Table 1) and the fitted Gaussian curves (sp-ICP-MS histograms) obtained under maximum sensitivity conditions. HR-TEM size distributions (Figures S1-S5) are provided for comparison. Uncertainty corresponds to 1 Standard Deviation. For comparison purposes, corresponding relative standard deviations are given in brackets.

TEM	This approach
16.4 ± 2.0 (12%)	16.7 ± 1.6 (10%)
24.4 ± 2.7 (11%)	24.1 ± 2.0 (8.4%)
27.4 ± 3.8 (14%)	27.3 ± 4.0 (15%)
39.6 ± 4.7 (12%)	39.7 ± 6.9 (17 %)
73.3 ± 9.6 (13%)	71.1 ± 8.8 (12%)

Table S3. Comparison of the relative errors and mean of the replicates experimentally obtained with the concentric nebulizer with low TE (2.5%) used throughout the work and a Total Consumption Nebulizer with high TE (100%). *Uncertainty corresponds to the standard error of the mean (k=2) of the TEM images (Figures S1-S7); **combined standard uncertainty comprising individual uncertainty and reproducibility of the triplicates (95 % confidence interval).

Diameter TEM*	Regular Nebulizer (low TE)		Total Consumption Nebulizer (high TE)	
	Diameter**	Error (%)	Diameter**	Error (%)
16.4 ± 0.3	16.7 ± 0.7	1.8	16.6 ± 1.7	1.2
27.4 ± 0.5	27.3 ± 0.9	0.4	27.2 ± 2.1	0.7
39.6 ± 0.7	39.7 ± 1.3	0.3	40.2 ± 2.9	1.5

Table S4. Relative error and mean of the replicates experimentally obtained using the approach developed for the 39.6 nm AuNPs using different dwell times. *Uncertainty corresponds the combined standard uncertainty comprising individual uncertainty and reproducibility of the n-replicates (95 % confidence interval, n=3).

Integration Time	Size*
5 ms	40.2 ± 4.2
3 ms	39.1 ± 4.1
1 ms	40.1 ± 4.7
0.5 ms	40.4 ± 4.1
0.1 ms	39.5 ± 4.6

Table S5. Relative error and mean of the replicates experimentally obtained using the approach developed for the 24.4 nm AuNPs using different dwell times. *Uncertainty corresponds the combined standard uncertainty comprising individual uncertainty and reproducibility of the n-replicates (95 % confidence interval, n=3).

Integration Time	Size*
5 ms	23.9 ± 2.5
3 ms	25.0 ± 2.6
1 ms	24.5 ± 2.8
0.5 ms	25.2 ± 3.3
0.1 ms	25.0 ± 2.9

Table S6. Relative error and mean of the replicates experimentally obtained using the approach developed for the 16.7 nm AuNPs using different dwell times. *Uncertainty corresponds the combined standard uncertainty comprising individual uncertainty and reproducibility of the n-replicates (95 % confidence interval, n=3).

Integration Time	Size*
5 ms	16.6 ± 1.2
3 ms	16.7 ± 1.5
1 ms	16.2 ± 1.6
0.5 ms	16.8 ± 1.7
0.1 ms	17.2 ± 2.5

Table S7. Mean of the three replicates experimentally obtained using the approach developed at different concentration levels. *Uncertainty corresponds to the standard error of the mean (k=2) of the TEM images (Figures S1-S7); **combined standard uncertainty comprising individual uncertainty and reproducibility of the triplicates (95 % confidence interval).

Diameter TEM (nm)*	Concentration (ppb)	Diameter (nm)**
27.4 ± 0.5	250 (1X)	27.1 ± 1.7
	500 (2X)	27.9 ± 2.9
	750 (3X)	27.6 ± 2.8
39.6 ± 0.7	170 (1X)	39.5 ± 2.8
	340 (2X)	40.5 ± 4.2
	510 (3X)	40.6 ± 4.2

Description of surface functionalization of AuNPS

AuNPs (18.7 nm) conjugated to Avidin

Avidin is a tetrameric binding protein (68 KDa), which is extensively used to facilitate further bioconjugations of the nanoparticles by affinity. Surface functionalization of AuNPs with avidin is carried out by a simple adsorption procedure. Citrate-based AuNPs were incubated with a large excess of Avidin to ensure surface saturation. The conjugated AuNPs were then purified to remove any protein excess.

AuNPs (24.4 nm) conjugated to PEG.

AuNPs were bioconjugated with 3 KDa PEG-thiolate modified with carboxyl groups. This ligand is typically used to render NPs with more stability in water media and biocompatibility. The sulfhydryl group is strongly attached to the Au atoms at the surface of the AuNP leaving the carboxyl groups at the other end of the PEG molecule oriented to the solution in a similar way as the originally present citrate molecule.

Protein Corona formation over different AuNPs (18.7, 39.6 and 73.3 nm)

Bovine Serum Albumin (BSA) was selected as model protein as its standard is highly characterized and it is very similar to the Human Serum Albumin, which is the most abundant protein in human serum. BSA was incubated with the AuNPs of different sizes for 24h under constant agitation at room temperature to generate the NP protein corona. In this studies the protein:metal ratio were kept high (i.e. 9500:1; BSA concentration 0.5%) and stable in order to produce a significant protein layer(s). For that purpose, the molar ratio BSA:AuNPs was adapted in each case: 1:30 in the case of the 18.7 nm AuNPs; 1:300 in the case of the 39.6 nm AuNPs and 1:1200 in the case of the 73.3 nm AuNPs. Resulting mixtures were not purified to mimic crude biological samples.
